# Association between non-alcoholic fatty liver and acute cerebral infarction: a protocol of systematic review and meta-analysis

**DOI:** 10.1097/MD.0000000000020351

**Published:** 2020-06-12

**Authors:** Ya-Juan Zhang, Wen-Juan Liu

**Affiliations:** Department of Neurology, First Affiliated Hospital of Jiamusi University, Jiamusi , China.

**Keywords:** acute cerebral infarction, association, non-alcoholic fatty liver

## Abstract

**Background:**

This study will systematically synthesize the evidence on the potential association between non-alcoholic fatty liver (NAFL) and acute cerebral infarction (ACI).

**Methods:**

We will propose literature search in electronic databases (MEDLINE, EMBASE, Cochrane Library, Scopus, Web of Science, WANGFANG, and China National Knowledge Infrastructure) from the source to March 1, 2020. There are no restrictions related to the language and publication status. Two review authors will separately carry out records selection, data extraction and study quality assessment. Any divisions will be solved by discussion with consulting a third review author. We will use RevMan 5.3 software to perform data analysis.

**Results:**

The present study will afford additional insight into the investigation the association between NAFL and ACI.

**Conclusion:**

The results of this study will provide helpful evidence to explore the association between NAFL and ACI.

Study registration number: INPLASY202040102.

## 1 Introduction

Acute cerebral infarction (ACI) is common type of severe neurological disease,^[1–4]^ which is characterized by neurological deficit syndrome caused by a sudden blood flow supply interruption to brain.^[5–8]^ Previous studies reported that patients with ACI have high incidence, disability, mortality, and recurrence rate,^[9–12]^ which result in tremendous impact of quality of life in such population.^[13–16]^ A variety of risk factors are reported to have close association with ACI, such as hypertension, smoking, ischemic heart disease, hyperlipidaemia, diabetes mellitus, carotid artery stenosis, atrial fibrillation, obesity, family history of stroke, physical inactivity and non-alcoholic fatty liver (NAFL).^[6,17–22]^ Several studies have reported that there is association between NAFL and ACI.^[22–27]^ However, there is not systematic review focusing on this topic. Thus, this study aims to systematically investigate the association between NAFL and ACI.

## 2 Methods

### 2.1 Objective

This study will aim to explore the association between NAFL and ACI systematically and comprehensively.

### 2.2 Study registration

This study has been registered on INPLASY202040102. It has been reported according to the guideline of preferred reporting items for systematic reviews and meta-analysis protocol statement.^[28]^

### 2.3 Inclusion criteria for study selection

#### 2.3.1 Type of studies

All potential case-controlled studies will be included, which identified the association between NAFL and ACI, regardless language and publication status limitations.

#### 2.3.2 Type of participants

In this study, the reports of all subjects with NAFL and ACI, or normal healthy participants will be included, regardless race, age, and sex.

#### 2.3.3 Type of exposures

All subjects in the experimental group had NAFL and ACI.

All participants in the control group were healthy without NAFL and ACI.

#### 2.3.4 Type of outcome measurements

Outcomes are severity of ACI (measured by National Institutes of Health Stroke Scale, or other scales), serum levels of glucose, triglycerides, total cholesterol, low density lipoprotein cholesterol, high-density lipoprotein cholesterol, creatinine, serum alanine aminotransferase, aspartate aminotransferase, and uric acid.

### 2.4 Data sources and search strategy

#### 2.4.1 Electronic searches

This study will comprehensively search electronic databases (MEDLINE, EMBASE, Cochrane Library, Scopus, Web of Science, WANGFANG, and China National Knowledge Infrastructure) from their sources to the March 1, 2020 without limitations related to the language and publication status. We have created search strategy sample for MEDLINE (Table 1), and have adapted similar search strategies for other electronic databases.

**Table 1:**
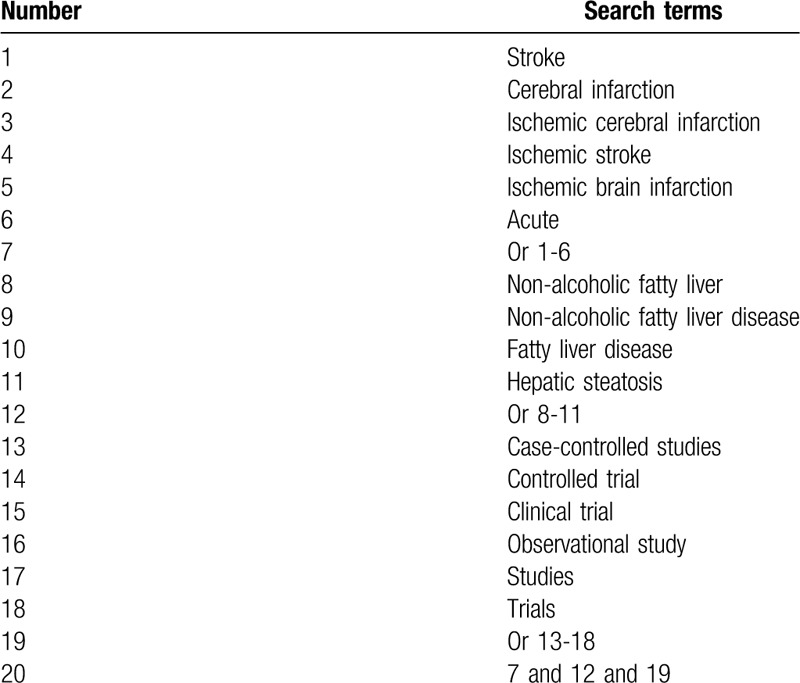
Search strategy for MEDLINE.

#### 2.4.2 Other resources

We will identify other sources, including conference abstracts and reference lists of included trials.

### 2.5 Data collection and analysis

#### 2.5.1 Selection of studies

Two review authors will independently examine all searched citations, and all duplicates will be removed. Titles/abstracts of all potential studies will be screened to exclude any irrelevant studies. Full papers of remaining trials will be carefully read against all inclusion criteria. Any disagreements will be arbitrated through discussion with the help of a third review author. The process of study selection will be presented in a PRISMA flow diagram.

#### 2.5.2 Data collection and management

Two review authors will independently collect data from included articles. Any discrepancies between 2 review authors will be solved through discussion with a third review author. We will collect study title, first author, year of publication, location, participant characteristics (such as age, gender, severity of NAFL and ACI), outcomes, results, conclusions, and conflict of interest.

#### 2.5.3 Dealing with missing data

Any insufficient or missing data will be obtained from original study authors. We will analyze available data using an intention-to-treat analysis if we cannot receive any reply.

### 2.6 Study quality assessment

Study quality of included case-controlled studies will be examined by 2 independent review authors using Newcastle-Ottawa Scale.^[29]^ Divisions between two review authors will be settled by discussion with a third review author.

### 2.7 Statistical analysis

#### 2.7.1 Data synthesis

This study will employ RevMan V.5.3 to pool and analyze all extracted outcome data. We will exploit dichotomous data by risk ratio and 95% confidence intervals (CIs). We will express continuous data by weighted mean difference or standardized mean difference and 95% CIs. We will use *I*^2^ test to check heterogeneity. The values of *I*^2^ are defined as follows: *I*^2^ ≤ 50% shows homogeneity, we will use a fixed-effects model, while *I*^2^ > 50% suggests apparent heterogeneity, and we will utilize a random-effects model. We will carry out a meta-analysis if homogeneity is examined. Otherwise, we will perform a subgroup analysis to investigate the possible sources of distinct heterogeneity.

#### 2.7.2 Subgroup analysis

If possible, a subgroup analysis will be performed according to the study characteristics, study quality, and outcomes.

#### 2.7.3 Sensitivity analysis

We will carry out a sensitivity analysis to check the robustness of study results by removing the low quality studies.

#### 2.7.4 Reporting bias

If over 10 studies are included, we will check reporting bias using a funnel plot and an Egger regression test.^[30]^

### 2.8 Ethics and dissemination

This study will only collect data from published studies, thus, no research ethic is needed.

We will submit this study on a peer-reviewed journal or a related conference meeting.

## 3 Discussion

To the best of our knowledge, this is the first study to investigate the association between NAFL and ACI. It will systematically and comprehensively search electronic databases, and other literature sources to avoid missing potential studies. This study will summarize the most recent eligible studies of the association between NAFL and ACI. The findings of this study will provide evidence to judge the association between NAFL and ACI, which may benefit for the clinical practice and future studies.

## Author contributions

**Conceptualization:** Wen-Juan Liu.

**Data curation:** Ya-Juan Zhang, Wen-Juan Liu.

**Formal analysis:** Ya-Juan Zhang.

**Funding acquisition:** Wen-Juan Liu.

**Investigation:** Wen-Juan Liu.

**Project administration:** Wen-Juan Liu.

**Resources:** Ya-Juan Zhang.

**Software:** Ya-Juan Zhang.

**Supervision:** Wen-Juan Liu.

**Validation:** Ya-Juan Zhang, Wen-Juan Liu.

**Visualization:** Ya-Juan Zhang, Wen-Juan Liu.

**Writing – original draft:** Ya-Juan Zhang, Wen-Juan Liu.

**Writing – review & editing:** Ya-Juan Zhang, Wen-Juan Liu.

## References

[1] Naess H Waje-Andreassen U Thomassen L Monitoring in acute cerebral infarction. Tidsskr Nor Laegeforen 2006;126:444–6. 16477281

[2] Shimosegawa E Actual diagnostic condition of acute cerebral infarction. Nihon Rinsho 2006;64: Suppl 8: 16–9. 17469528

[3] Strong K Mathers C Bonita R Preventing stroke: saving lives around the world. Lancet Neurol 2007;6:182–7. 17239805 10.1016/S1474-4422(07)70031-5

[4] Chen WW Gao RL Liu LS Summary of China Cardiovascular report 2015. Chin J Circul 2016;31:521–8.

[5] Kara H Akinci M Degirmenci S High-sensitivity C-reactive protein, lipoprotein-related phospholipase A2, and acute ischemic stroke. Neuropsychiatr Dis Treat 2014;10:1451–7. 25125979 10.2147/NDT.S67665 PMC4130710

[6] Dong XL Xu SJ Zhang L Serum resistin levels may contribute to an increased risk of acute cerebral infarction. Mol Neurobiol 2017;54:1919–26. 26899574 10.1007/s12035-016-9751-3

[7] Lv G Wang GQ Xia ZX Influences of blood lipids on the occurrence and prognosis of hemorrhagic transformation after acute cerebral infarction: a case-control study of 732 patients. Mil Med Res 2019;6:2. 30665465 10.1186/s40779-019-0191-z PMC6341695

[8] Paiboonpol S Hyperglycemia in acute cerebral infarction. J Med Assoc Thai 2006;89:614–8. 16756045

[9] Roth GA Johnson CO Nguyen G Methods for estimating the global burden of cerebrovascular diseases. Neuroepidemiology 2015;45:146–51. 26505980 10.1159/000441083

[10] Mozaffarian D Benjamin EJ Go AS Executive summary: Heart disease and stroke statistics–2016 update: a report from the American Heart Association. Circulation 2016;133:447–54. 26811276 10.1161/CIR.0000000000000366

[11] Zhao XJ Li QX Liu TJ Predictive values of CSS and NIHSS in the prognosis of patients with acute cerebral infarction: a comparative analysis. Medicine (Baltimore) 2018;97:e12419. 30278519 10.1097/MD.0000000000012419 PMC6181457

[12] Ono H Nishijima Y Ohta S Hydrogen gas inhalation treatment in acute cerebral infarction: A randomized controlled clinical study on safety and neuroprotection. J Stroke Cerebrovasc Dis 2017;26:2587–94. 28669654 10.1016/j.jstrokecerebrovasdis.2017.06.012

[13] Sun Z Xu Q Gao G Clinical observation in edaravone treatment for acute cerebral infarction. Niger J Clin Pract 2019;22:1324–7. 31607719 10.4103/njcp.njcp_367_18

[14] Li L Ren S Hao X Efficacy of minimally invasive intervention in patients with acute cerebral infarction. J Cardiovasc Pharmacol 2019;73:22–6. 30540689 10.1097/FJC.0000000000000625

[15] Zhen X Zheng Y Hong X Physiological ischemic training promotes brain collateral formation and improves functions in patients with acute cerebral infarction. Front Neurol 2016;7:235. 28066319 10.3389/fneur.2016.00235 PMC5177612

[16] Liu S, Wang K, Duan X, et al. Efficacy of danshen class injection in the treatment of acute cerebral infarction: a Bayesian network meta-analysis of randomized controlled trials. Evid Based Complement Alternat Med. 2019; 2019:5814749. 10.1155/2019/5814749 PMC6377994 30854011

[17] Ohta Y Takao Y Harada K Metabolic syndrome is a risk factor for acute cerebral infarction in a younger elderly Kurashiki population. J Stroke Cerebrovasc Dis 2012;21:231–9. 20851624 10.1016/j.jstrokecerebrovasdis.2010.07.003

[18] Wang R Zeng J Wang F Risk factors of hemorrhagic transformation after intravenous thrombolysis with rt-PA in acute cerebral infarction. QJM 2019;112:323–6. 30566606 10.1093/qjmed/hcy292

[19] Xu X Li C Wan T Risk factors for hemorrhagic transformation after intravenous thrombolysis in acute cerebral infarction: a retrospective single-center study. World Neurosurg 2017;101:155–60. 28185970 10.1016/j.wneu.2017.01.091

[20] Hori YS Kodera S Sato Y Eosinopenia as a predictive factor of the short-term risk of mortality and infection after acute cerebral infarction. J Stroke Cerebrovasc Dis 2016;25:1307–12. 26971036 10.1016/j.jstrokecerebrovasdis.2015.12.007

[21] Zhang F Li X Dong Q Risk of acute cerebral infarction and plasma asymmetrical dimethylarginine and homocysteine levels: a clinical correlation analysis of Chinese population. J Stroke Cerebrovasc Dis 2014;23:2225–32. 25169826 10.1016/j.jstrokecerebrovasdis.2014.04.001

[22] Alkagiet S Papagiannis A Tziomalos K Associations between nonalcoholic fatty liver disease and ischemic stroke. World J Hepatol 2018;10:474–8. 30079133 10.4254/wjh.v10.i7.474 PMC6068844

[23] Abdeldyem SM Goda T Khodeir SA Nonalcoholic fatty liver disease in patients with acute ischemic stroke is associated with more severe stroke and worse outcome. J Clin Lipidol 2017;11:915–9. 28579247 10.1016/j.jacl.2017.04.115

[24] Tziomalos K Giampatzis V Bouziana SD Association between nonalcoholic fatty liver disease and acute ischemic stroke severity and outcome. World J Hepatol 2013;5:621–6. 24303090 10.4254/wjh.v5.i11.621 PMC3847945

[25] Ying I Saposnik G Vermeulen MJ Nonalcoholic fatty liver disease and acute ischemic stroke. Epidemiology 2011;22:129–30. 21150361 10.1097/EDE.0b013e3181feb50a

[26] Chen QQ Liang CC Yang LJ Clinical analysis of Uyghur patients with acute ischemic cerebral infarction and nonalcoholic fatty liver disease. J Clin Hepatol 2018;34:1259–63.

[27] Chen MM Zhang SY Chen GY Clinical analysis of the correlation between non-alcoholic fatty liver disease and acute cerebral infarction. J Pract Hepatol 2015;18:150–5.

[28] Shamseer L Moher D Clarke M PRISMA-P Group. Preferred reporting items for systematic review and meta-analysis protocols (PRISMA-P) 2015: elaboration and explanation. BMJ 2015;349:g7647. 10.1136/bmj.g7647 25555855

[29] Stang A Critical evaluation of the Newcastle-Ottawa scale for the assessment of the quality of nonrandomized studies in meta-analyses. Eur J Epidemiol 2010;25:603–32. 20652370 10.1007/s10654-010-9491-z

[30] Deeks JJ Macaskill P Irwig L The performance of tests of publication bias and other sample size effects in systematic reviews of diagnostic test accuracy was assessed. J Clin Epidemiol 2005;58:882–93. 16085191 10.1016/j.jclinepi.2005.01.016

